# Analysis of the C2H2 Gene Family in Maize (*Zea mays* L.) under Cold Stress: Identification and Expression

**DOI:** 10.3390/life13010122

**Published:** 2022-12-31

**Authors:** Sinan Li, Yunlong Li, Quan Cai, Xin Li, Yan Sun, Tao Yu, Jianfei Yang, Jianguo Zhang

**Affiliations:** Heilongjiang Academy of Agricultural Sciences, Harbin 150086, China

**Keywords:** maize (*Zea mays* L.), cold stress, C2H2 gene family

## Abstract

The C2H2 zinc finger protein is one of the most common zinc finger proteins, widely exists in eukaryotes, and plays an important role in plant growth and development, as well as in salt, low-temperature, and drought stress and other abiotic stress responses. In this study, C2H2 members were identified and analyzed from the low-temperature tolerant transcriptome sequencing data of maize seedlings. The chromosome position, physical and chemical properties, evolution analysis, gene structure, conservative motifs, promoter cis elements and collinearity relationships of gene the family members were analyzed using bioinformatics, and the expression of the ZmC2H2 gene family under cold stress was analyzed by fluorescent quantitative PCR. The results showed that 150 members of the C2H2 zinc finger protein family were identified, and their protein lengths ranged from 102 to 1223 bp. The maximum molecular weight of the ZmC2H2s was 135,196.34, and the minimum was 10,823.86. The isoelectric point of the ZmC2H2s was between 33.21 and 94.1, and the aliphatic index was 42.07–87.62. The promoter cis element analysis showed that the ZmC2H2 family contains many light-response elements, plant hormone-response elements, and stress-response elements. The analysis of the transcriptome data showed that most of the ZmC2H2 genes responded to cold stress, and most of the ZmC2H2 genes were highly expressed in cold-tolerant materials and lowly expressed in cold-sensitive materials. The real-time quantitative PCR (qRT-PCR) analysis showed that ZmC2H2-69, ZmC2H2-130, and ZmC2H2-76 were significantly upregulated, and that ZmC2H2-149, ZmC2H2-33, and ZmC2H2-38 were significantly downregulated. It is hypothesized that these genes, which function in different metabolic pathways, may play a key role in the maize cold response. These genes could be further studied as candidate genes. This study provides a theoretical reference for further study on the function analysis of the maize C2H2 gene family.

## 1. Introduction

Maize (*Zea mays* L.) is a tropical crop, and its suitable growth temperature is 25–28 °C. If the temperature is too low, it will affect its growth and development and even affect the yield. The northern early spring corn area is an important corn production area and commodity food base in China. However, due to the specificity of the geographical location and environmental conditions, a low temperature during spring emergence is an important abiotic stress that affects the quality of seedlings and severely restricts the yield and quality of maize. It has been reported that for every 0.7 °C decrease during the reproductive period, the developmental cycle of maize is prolonged by 7 days, along with an 8% yield reduction [1]. Improving cold tolerance at the seedling stage will provide far-reaching implications for maize production at high latitudes [2]. Therefore, there is a need to address the issues related to cold stress in maize from the perspective of molecular biology.

The transcription of genes is essential for maintaining life, as it changes the expression of proteins, and it influences their activities, while an involuted regulatory system of transcription controls the expression of encoding proteins at suitable opportunities, especially transcription factors [3,4]. Zinc finger protein (ZFP) transcription factors, a large protein family, participate in the various stages of plant growth and development as well as stresses (including biotic and abiotic stresses) [5]. The domain of ZFPs is highly conserved and contains a consensus sequence of CX2-4CX3FX5LX2HX3-5H and approximately 20–30 amino acids [6]. The structures of ZFPs show diversity, which are varied and divided into several classes according to the positions and numbers of cysteine (Cys) and histidine (His) with zinc ion residues, such as C2H2, C2HC, C2HC5, C2C2, C3HC4, C4, C4HC3, and C6 [7,8,9]. While the functions of ZFPs also show diversity, it has been reported that some ZFPs act as key regulators of membrane association, bind to nucleic acids (including DNA/RNA) and recognize protein interactions [8]. The *C2H2* zinc finger, as an important class of ZFPs, mainly exists in eukaryotes, the structure of which has the characteristic of one to four zinc fingers motif(s) found in the *C2H2* finger and combined with Cys and His residues [10,11]. In the structure, the domain contains one α-helix in the C-terminus and two β-strands in the N-terminus, and the zinc atom is sandwiched between the α-helix and two β-strands, forming a tetrahedral structure [5,10,12]. Notably, plant-specific *C2H2* members have different lengths between the two zinc fingers compared with other eukaryotic species [13].

For *C2H2*, it has been reported that some members function as a key regulator in many biological processes, such as regulating membrane association, binding to nucleic acids (including DNA/RNA), and recognizing protein interactions [14]. These *C2H2* members also participate in the growth and development of many plants and, in some organs and structures, *C2H2* members have been found to be involved in processes [7]. The *EFP1* gene was the first gene identified in plants, and it has a function in petal development in petunia [15]. *Zinc finger protein 3* is a member of *C2H2* found in Arabidopsis, which participates in seed germination and plant development and could interfere with light signaling [16]. Furthermore, *MAZ1* is a *C2H2* member isolated from Arabidopsis and is essential for intine and exine formation [17]. *Nonstop glumes 1* is a gene found in rice (*Oryza sativa*) that encodes the C2H2 protein, and it has a higher expression in the organ primordia and regulates spikelet development through transgenic plants and mutants [18]. Hair is a gene found in tomato (*Solanum lycopersicum*) encoding the C2H2 protein, which can regulate multicellular trichome formation [19]. The *ZFP5* is a *C2H2* member found in Arabidopsis that is associated with ethylene signaling and regulating root hair development [20]. Furthermore, *C2H2* members have also been reported to play an extensive role in plant tolerance responses to various biotic and abiotic stresses, especially cold stress [21,22]. Indeed, *ZAT12* is a transcription factor of the *C2H2* family, which can control 15 cold-suppressed genes’ expression and regulates cold acclimation in Arabidopsis [23]. The soybean zinc finger protein (*SCOF-1*) is a transcription factor that regulates cold tolerance, which the overexpressing *SCOF-1* in transgenic Arabidopsis and tobacco (*Nicotiana tabacum*) plants exhibited, inducing the COR (cold-regulated) genes’ expression and enhancing cold tolerance [24]. A novel *C2H2* member from tomato (*SlCZFP1*) has been reported to be able to induce cold-responsive genes’ expression and enhance cold tolerance in transgenic Arabidopsis and rice, while the overexpression of *MaC2H2-2* and *MaC2H2-3* (C2H2 members in banana) repressed the cold signaling pathway significantly [25,26]. *GmZF1* is a *C2H2* member in soybean (*Glycine max*) that responds to the cold-stress-related gene *COR6.6* in transgenic plants and regulates cold stress resistance [27]. All of the current studies reveal that *C2H2* members participate in cold stress [6].

As an important transcription factor, *C2H2* members have been identified in Arabidopsis [5], rice (*Oryza sativa*), durum wheat (*Triticum turgidum Durum*), soybean (*Glycine max*), potato (*Solanum tuberosum*), and sorghum (*Sorghum bicolor*) [28,29,30], but few studies have identified it in maize (*Zea mays*). Maize is an important crop that is domesticated from wild grass (*Z. mays* subsp. *Parviglumis*), and cold stress can reduce both the seed germination rate and the vigor of the seedlings [31,32,33]. The cell membranes become impaired, resulting in the loss of cellular components and a permanent change in the chemical properties [34]. The cells of the seedlings are irreversibly damaged under cold stress [35], which can reduce the height, root length, chlorophyll content, and net photosynthetic rate of the plant and directly lead to stunted seedling growth, wilting and necrosis of leaves, and even plant death [36,37]. Cold can also increase the chances of infection by soil bacteria, affecting plant health [38]. In this study, we identified *C2H2* gene family members from the maize genome and analyzed the bioinformatics and relative gene expression of the *C2H2* gene family members, which provides the basis for a subsequent study of maize *C2H2* members.

## 2. Materials and Methods

### 2.1. Bioinformatics Analysis of the C2H2 Gene Family

The reference genome (including DNA, RNA, cDNA and protein sequence) was provided by the Esembl plants database, and the reference version was Zm-B73-REFERENCE-NAM-5.0 (https://plants.ensembl.org/Zea_mays/Info/Index) (accessed on 10 October 2022.)Hmmsearch and hmmbuild were used for the identification with a Perl script, and the *C2H2* domain (PF00096) was provided by PFAM (pfam.xfam.org). The members of the *C2H2* domain were confirmed using SMART software, and the duplicates were removed [39]. The *C2H2* members of maize (*Zea mays*) were named according to their location on chromosome, which was shown on the Esembl plants database by Tbtools software [40]. MEGA X was used to build an evolutionary tree using neighbor-joining (NJ) methods and the dayhoff+g model predicted by MEGAX, and the 1000 bootstrap was used to calculate the evolutionary relationship of the *C2H2* members in maize [41]. The motif of the *C2H2* members was identified using MEME software, and the length of each motif was 10–50 amino acids, while the value was less than 1 × 10^−10^ [42]. The gene structure of the *C2H2* members was analyzed using Gene Structure Display Server software, and information on the DNA, RNA, and cDNA was also provided by the Esembl plant database [43]. The cis-acting elements were identified using PlantCARE, and the function of each cis-acting element was predicted by PlantCARE [44]. MCScanX software was used to calculate the collinear pairs of the C2H2 members using Perl commands [45].

### 2.2. Plant Materials and Treatments

The materials were T641, a cold-tolerant inbred line, and SX641, a cold-sensitive inbred line, which were screened in the northern region of Heilongjiang Province in the early stage. In this study, the cold-tolerant maize inbred line was named CT (cold tolerance), and the cold-sensitive maize inbred line was named CS (cold-sensitive).

The maize seeds were sown in seed culture boxes and placed in a light incubator for germination at 26 °C with 16 h of light, 8 h of darkness, and regular watering. The material was treated after 15 days of incubation until the three-leaf stage (V2).

Each material was treated at 4 °C for 24 h. The control material remained incubated at 26 °C, and the light and watering conditions were unchanged. The treated samples were named CT24 and CS24, and the controls were named CS and CS24. There were four samples in total, and each sample had three biological replicates. The test materials were selected from the leaves, which were quickly treated with liquid nitrogen and then preserved on dry ice, and the transcriptome sequencing was performed by a sequencing company.

### 2.3. RT-qPCR Analysis of The Candidate Genes

Real-time quantitative PCR (RT-qPCR) was performed using the cDNA samples returned by the sequencing company. The RT-qPCR primers were designed using Primer Premier v6.0 (http://www.premierbiosoft.com/primerdesign/index.html). The Actin 4 gene of maize was selected as the internal reference gene. The RT-qPCR program uses a Light Cycler 480 system (Roche, Roche Diagnostics, Basel, Switzerland) and a 2× ChamQ Universal SYBR qPCR Master Mix Kit (Vazyme, Q711, Vazyme biotech, Nanjing, China). Each RT-qPCR was biologically repeated three times. The relative expression of the candidate genes was calculated using the following formula:

Relative expression = 2^∆∆Ct^, {∆∆Ct = [Ct_2_(Zm target genes) − Ct_2_(ACTIN4)] − [Ct_1_(Zm target genes) − Ct_1_ (ACTIN4)]} (1)

## 3. Results

### 3.1. Bioinformatics Analysis of the C2H2 Gene Family

#### 3.1.1. Identification of C2H2 Members

**Figure 1:**
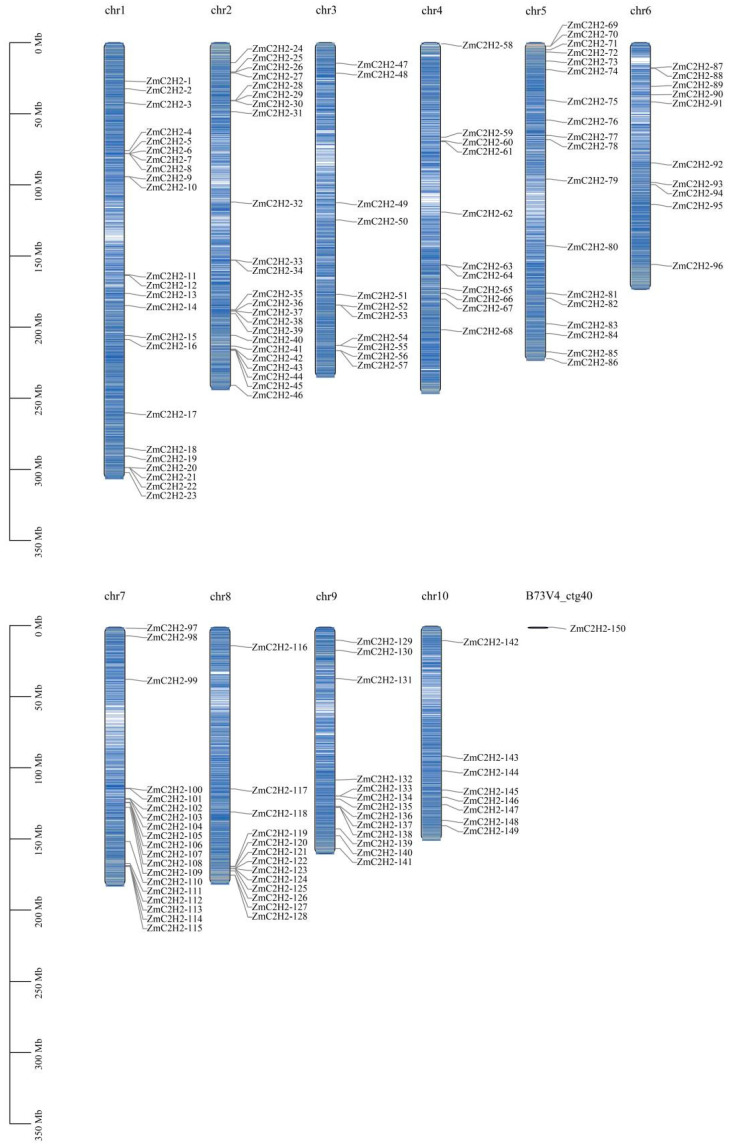
Location of the *ZmC2H2* members. The black line on the left measures the chromosome’s length, each column of different lengths represents chromosomes Chr1 to Chr11, and “B73V4_ctg40” represents the splices. The density of the line of the column represents the density of the genes on the chromosome.

Through hmmsearch and hmmbuild, 166 members were identified, and after removing the duplicates, 150 members were left that could be used as C2H2 members in maize (*Zea mays*); these were then named from *ZmC2H2-1* to *ZmC2H2-150*. The location information is shown in Figure 1. The *ZmC2H2* members covered all of the chromosomes of the reference genome, while one *ZmC2H2* member (*ZmC2H2-150*) existed on the splice “B73V4_ctg40”, where Chr 1 and Chr 2 had the highest numbers (23). The number of *ZmC2H2* members on Chr 10 was the lowest (8). Detailed information is provided in Table S1. The characteristics of the *ZmC2H2* members showed great variation, where the protein length was from 102 to 1223 bp. The maximum value of the molecular weight of *ZmC2H2s* was 135,196.34, while the lowest was 10,823.86. The isoelectric point of *ZmC2H2s* was between 33.21 and 94.1, while the aliphatic index was 42.07–87.62. The above results provided the basic properties of *ZmC2H2s*.

#### 3.1.2. Evolution Analysis of the C2H2 Members

**Figure 2:**
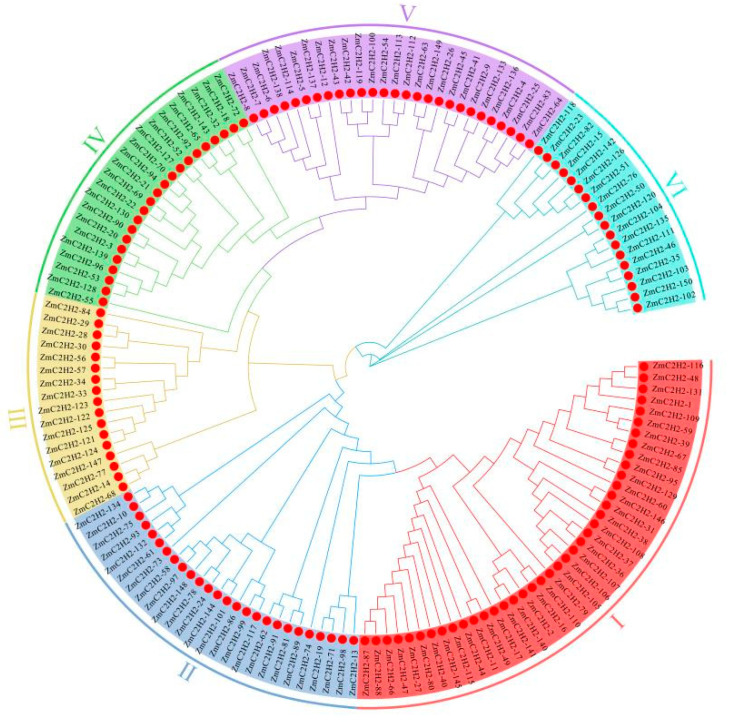
Evolution of the *C2H2* members. The six different colored blocks on the outer ring represent different subfamilies.

Six subfamilies were divided among the protein sequence’s fourteen members using MEGA X, and the method of division was as per that for *C2H2* members in a previous report [46]. Subfamily I had the most members of *ZmC2H2*, at 40 members, while only 17 members were in subfamily III (lowest number). Subfamily II and V had a similar number of *ZmC2H2* members, at 26 and 27, respectively (Figure 2).

#### 3.1.3. Motifs and Gene Structure of the ZmC2H2s

**Figure 3:**
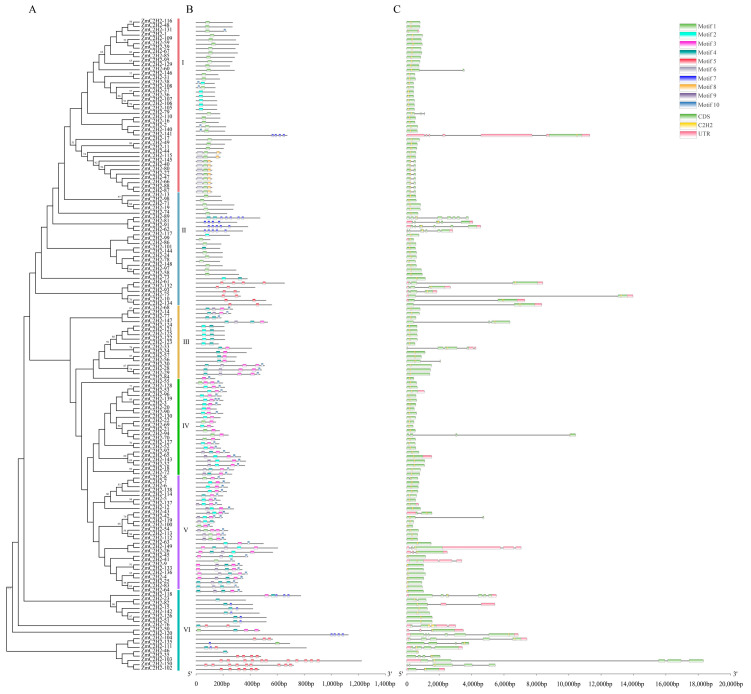
Motifs and gene structure of the *ZmC2H2* members. (**A**) Evolution of the *ZmC2H2s*, where the 5 colors represent the different subfamilies; (**B**) motifs of the *ZmC2H2s*, where the 10 different colored squares represent the different motifs; (**C**) gene structure of the *ZmC2H2s*, where the green squares represent the CDS region, the pink squares represent the UTR region, and the yellow squares represent the structure of the *C2H2*.

The motifs were identified in *ZmC2H2*, and the kinds and order, according to the evolutionary relationships, are shown in Figure 3A and Table S2. Each subfamily of *ZmC2H2* members had similar kinds of motifs. Motif 5 was only found in subfamily II and subfamily VI, and motif 4 did not exist in subfamilies I and II, while motif 6 was only found in subfamily I. The gene structures of the *ZmC2H2s* were also analyzed, and the results are shown in Figure 3C, where the structure of the different *ZmC2H2s* showed great variation. Here, *ZmC2H2-103* had the longest length, and members of the same subfamily had a similar gene structure according to the analysis.

#### 3.1.4. Cis-Acting Elements of the ZmC2H2s

The cis-acting elements in the *ZmC2H2* members were identified and predicted using a function in the PlantCare software. The results show the order according to the evolutionary relationship (Table S3). The elements were divided into four kinds according to their function. The hormone-related cis-acting elements (such as ABRE, AuxRR-core, CGTCA-motif, GARE-motif, P-box, TATC-box, TCA-element, TGACG-motif, and TGA-element) were related to plant hormones, which revealed that some *ZmC2H2* members had the function of responding to hormones. The stress-related elements (such as MBS, LTR, GC-motif, WUN-motif, and ARE) were connected with environmental stresses, which suggests that some members might regulate stress. The light-related elements (including Box 4, ATCT-motif, G-Box, ACE, AE-box, Sp1, GT1-motif, TCCC-motif, GATA-motif, I-box, chs-CMA2a, and TCT-motif) were the light-responsive elements, while the blue elements had a relationship with cell cycle regulation, which illustrates that some members might have a function in the seedling stage.

#### 3.1.5. Collinearity and Ka/Ks Analysis of the ZmC2H2s

**Figure 4:**
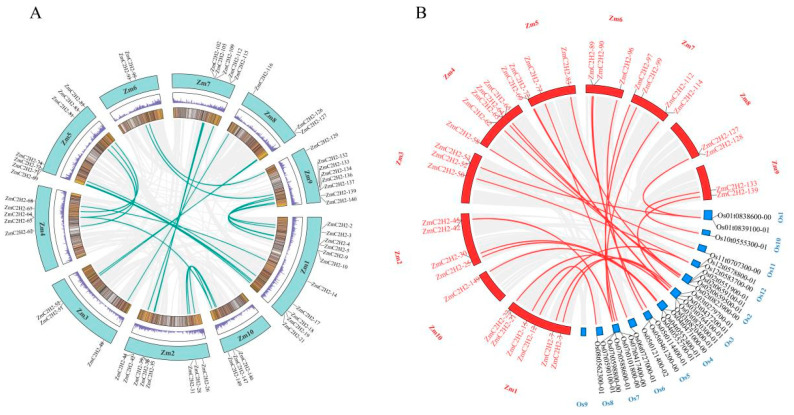
Collinearity of the *ZmC2H2* members. (**A**) Collinearity of the *ZmC2H2s*, where the two circles in the middle represent the gene density, the green lines represent collinear gene pairs, and the gray background represents all of the collinear pairs of genes in maize; (**B**) collinearity of *ZmC2H2s* with members in rice, where the red lines represent collinear gene pairs, and the gray background represents all of the collinear pairs of genes between two species.

The collinear pairs were calculated in this study, and the results are shown in Figure 4. In the collinearity of the ZmC2H2s, 28 pairs of *ZmC2H2* members had collinearity (Figure 4A). Compared with rice, there were 34 pairs of collinear relationships with *ZmC2H2s*, which reveals that these collinear members might have a similar function (Figure 4B). In the Ka/Ks analysis of the *ZmC2H2s*, 28 pairs of *ZmC2H2s* produced a Ka/Ks relationship, and only the Ka/Ks value of one pair of ZmC2H2 members (*ZmC2H2-42* and *ZmC2H2-112*) was more than one, and this pair of genes might produce a positive evolution, while the other pairs of *ZmC2H2* members showed pure evolution (Table S4).

### 3.2. RNA-Seq Analysis of the ZmC2H2s

**Figure 5:**
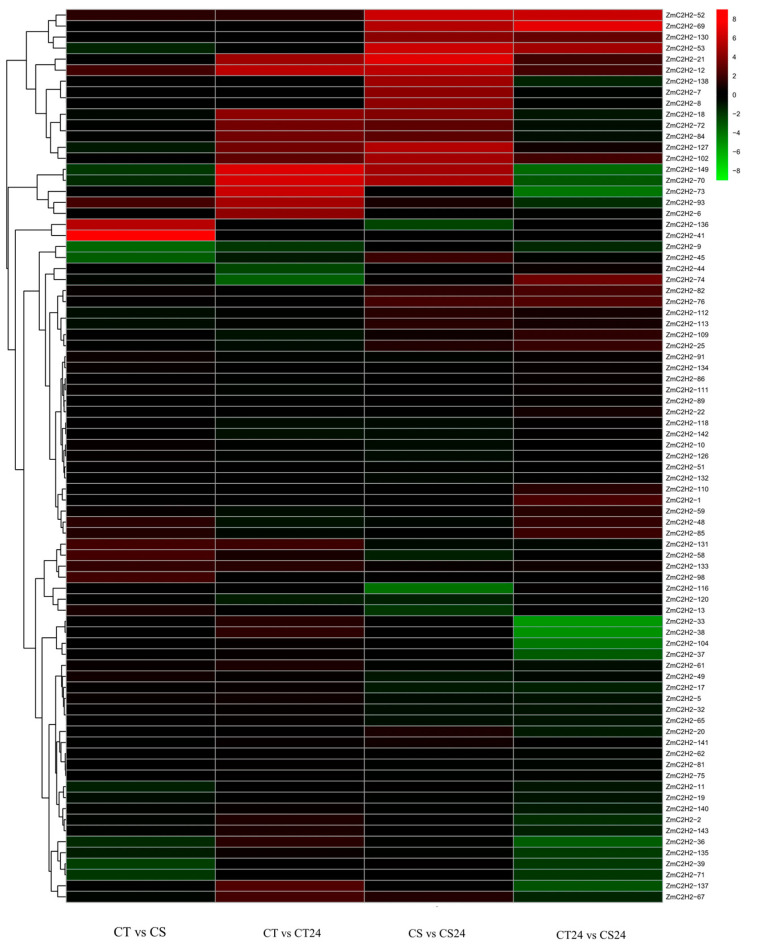
Heat map of the expression of the C2H2 gene family in maize under cold stress.

The transcriptome data of the cold-tolerant materials and the cold-sensitive materials under cold stress were analyzed to obtain a heat map of the expression pattern of the ZmC2H2 gene family (Figure 5). The results show that, under cold stress, the expression of the ZmC2H2 gene was significantly different between the different materials and between the different treatment times of the same material, indicating that the ZmC2H2 gene can be induced by low temperatures. Under cold stress, 69 genes in the ZmC2H2 gene family were not expressed, and the other 81 genes were expressed. The change in the expression of the ZmC2H2 genes in CT was more significant than that in CS, and the expression of the up- and downregulated genes in CT24 was relatively higher.

### 3.3. The Expression of the ZmC2H2s under Cold Stress

**Figure 6:**
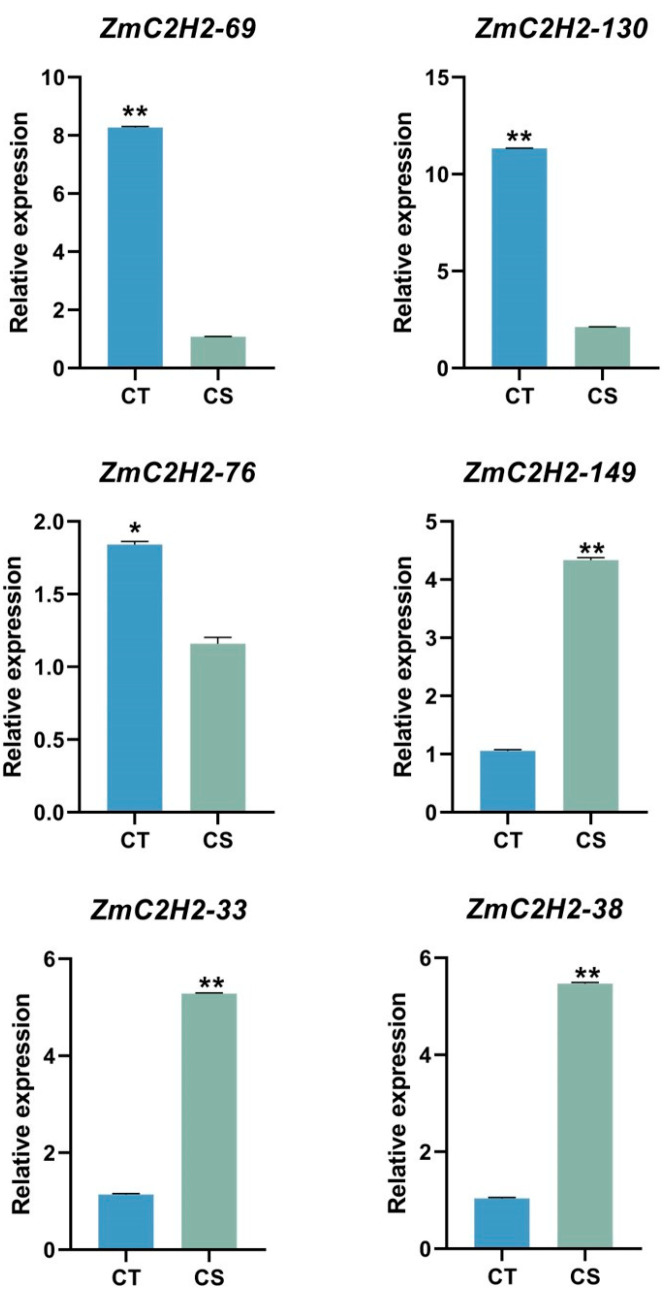
Relative expression of the candidate genes by RT-qPCR. Here, * denotes a significant difference between the extreme material and others (*p* < 0.05). ** denotes a extremely significant difference between the extreme material and others (*p* < 0.05).

In order to verify the expression of the *C2H2* gene family under cold stress, we selected the following three candidate genes involved in different pathways: auxin response gene (*ZmC2H2-69* and *ZmC2H2-130*); exogenous stress response gene (*ZmC2H2-76* and *ZmC2H2-149*); and light response gene (*ZmC2H2-33* and *ZmC2H2-38*). From the results, we can see that the expression of the genes changed before and after the treatment of the leaves. The results show that *ZmC2H2-69*, *ZmC2H2-130*, and *ZmC2H2-76* were significantly upregulated, and that *ZmC2H2-149*, *ZmC2H2-33*, and *ZmC2H2-38* were significantly downregulated (Figure 6). It is hypothesized that these genes, which function in different metabolic pathways, may play a key role in the maize cold response, and can be further investigated as candidate genes.

## 4. Discussion

Zinc finger proteins are one of the largest families of transcription factors in plants, and C2H2-type zinc finger protein transcription factors are the most studied zinc finger proteins, which play an important role in plant growth and development as well as in abiotic stress response, such as high salt, low temperatures, and drought. These *C2H2* members have been identified in some species, such as potato (*Solanum tuberosum*), alfalfa (*Medicago sativa*), Brassica rapa, Camellia sinensis, and ginseng (*Panax ginseng*), and the different species showed a wide number of changes [47,48,49]. There were 218 C2H2 members in alfalfa, and these members had 337 individual *C2H2* motifs (*Medicago truncatula*); 134 *C2H2* members were identified in Camellia sinensis, which were distributed on 15 chromosomes randomly [48,50]; 79 C2H2 proteins were identified in potato (*Solanum tuberosum*) through hmmsearch and BLASTP in the genome database [47]. In sorghum (*Sorghum bicolor*), 145 members were identified and distributed on 10 chromosomes [30]. In this study, 150 members were used as the *C2H2* members in maize (*Zea mays*), which covered all of the chromosomes of the reference genome and the splice “B73V4_ctg40”. There might be several reasons for this phenomenon. On the one hand, the size of the reference genome varies greatly, which directly leads to a number of the genes showing variations; even different reference genome versions of the same species lead to inconsistent gene family members [51,52]. Another aspect is the replication and expansion of the members in the process of evolution [53]. Exploring the evolution of the *C2H2* members could help in understanding the origin and history of the genes, which is helpful for analyzing and concluding the function of the genes [54]. The evolutionary analysis of the *C2H2* members in maize (*Zea mays*) also led to similar results; *ZmC2H2* members were divided into six subfamilies, and the number of subfamilies was similar to the *C2H2* members in Arabidopsis (six) [46]. These results indicate that the evolution of *C2H2* members might be completed before monocotyledon and dicotyledon differentiate, and that the Gramineae crop (*Maize*) and the model dicotyledon crop (*Arabidopsis*) had a similar evolutionary structure [55]. In addition, some *C2H2* members in cucumber (*Cucumis sativus*) and ginseng (*Panax ginseng*) also had six subfamilies, which reveals the accuracy of the *C2H2* evolution [49,55].

The results of the motifs of the *ZmC2H2* members showed that motifs 1–4 had a conserved sequence (“QALGGH”), which were plant-specific motifs, and these motifs were found in cucumber, Arabidopsis, durum wheat (*Triticum turgidum*) and rice (*Oryza sativa*) [12,28,56,57]. For the gene structure, the *C2H2* members had a similar number of exons, and the motif compositions were classified as being in the same subgroup; for those members in the same subgroup, this led to function transcription factors for protein interaction, transcriptional activity, and DNA binding [58]. The gene structure of the *ZmC2H2s* showed that each subgroup’s members had a similar gene structure. The cis-acting elements of the *ZmC2H2* members showed that they had functions related to hormones, stress, and seed germination. As a transcription factor family, *C2H2* members have been reported to have a connection with abiotic stresses, such as cold, heat, and drought [59,60]. Moreover, the cis-acting elements also contain hormone-related cis-acting elements, such as salicylic acid (SA), jasmonic acid (JA), ethylene (ET), and abscisic acid (ABA), in the *C2H2* members’ promoter in poplar (*Populus trichocarpa*) and ginseng (*Panax ginseng*) [49,61]. This reveals that the *C2H2* members are connected with hormones. The results of the colinear analysis of the *ZmC2H2* members had 34 pairs of collinearity, and these pair members had similar functions or jointly participated in regulating a certain metabolism, such as potato (*Solanum tuberosum*) [47]. The Ka/Ks showed that the *ZmC2H2s* were conservative in terms of evolutionary expansion, and most members displayed purity evolution, while the *C2H2* family has been considered to be a conservative gene family by predecessors [62,63]. All this detailed information help us to better understand and screen for appropriate *C2H2* gene family members in maize.

From the results of the RT-qPCR, it can be seen that the expression level of the candidate genes under cold stress changed significantly, and the change trend in the expression was significantly higher than that for the cold-sensitive materials. It is speculated that *ZmC2H2* genes feel low temperature faster in cold-resistant materials than in cold-sensitive materials. Cold-resistant materials may accelerate the transmission of low-temperature signals by increasing the rapid expression of *ZmC2H2* genes and inducing the expression of downstream cold-response genes. Therefore, the plant shows strong cold resistance. Its expression mode is similar to that of *SiC2H2-78* in millet [64], and its expression was significantly higher than that of the cold-sensitive materials, indicating that cold-resistant materials may resist low-temperature damage by increasing the expression of these genes, indicating that these genes play a positive role in regulating cold stress. Next, our subsequent studies will use the above candidate genes as the main entry point for overexpression or gene editing to generate new materials for cold-stressed maize, which will be used to analyze the performance and function of the candidate genes under cold stress and then to elucidate their molecular mechanisms.

## 5. Conclusions

In this study, 150 maize C2H2-ZFP genes were identified and divided into four groups (I–IV). Chromosomal location revealed that the 150 maize C2H2-ZFP genes were distributed in all maize chromosomes. The characteristics of the ZmC2H2 members showed great variation in the protein length, molecular weight, and isoelectric point, while the aliphatic index had a very large span. The expression analysis of the six selected ZmC2H2 genes in response to cold stress by RT-qPCR indicated that ZmC2H2 genes may be involved in different signaling pathways to modify maize resistance to cold stress. These results provide valuable information for future studies on the function of maize C2H2 genes.

## Data Availability

No New data.

## Supplementary Materials

The following supporting information can be downloaded at: https://www.mdpi.com/article/10.3390/life13010122/s1, Table S1: ZmC2H2 members, Table S2: The motifs of ZmC2H2, Table S3: The order according to the evolutionary relationship, Table S4: Ka/Ks analysis of the ZmC2H2s.
